# Comparative studies of IR spectra of deprotonated serine with classical and thermostated ring polymer molecular dynamics simulations

**DOI:** 10.1063/4.0000124

**Published:** 2021-09-14

**Authors:** V. S. Sandeep Inakollu, Haibo Yu

**Affiliations:** 1 School of Chemistry and Molecular Bioscience, University of Wollongong, Wollongong, New South Wales 2522, Australia; 2 Molecular Horizons, University of Wollongong, Wollongong, New South Wales 2522, Australia; 3 Illawarra Health and Medical Research Institute, Wollongong, New South Wales 2522, Australia

## Abstract

Here we report the vibrational spectra of deprotonated serine calculated from the classical molecular dynamics (MD) simulations and thermostated ring-polymer molecular dynamics (TRPMD) simulation with third-order density-functional tight-binding. In our earlier study [Inakollu and Yu, “A systematic benchmarking of computational vibrational spectroscopy with DFTB3: Normal mode analysis and fast Fourier transform dipole autocorrelation function,” J. Comput. Chem. **39**, 2067 (2018)] of deprotonated serine, we observed a significant difference in the vibrational spectra with the classical MD simulations compared to the infrared multiple photon dissociation spectra. It was postulated that this is due to neglecting the nuclear quantum effects (NQEs). In this work, NQEs are considered in spectral calculation using the TRPMD simulations. With the help of potential of mean force calculations, the conformational space of deprotonated serine is analyzed and used to understand the difference in the spectra of classical MD and TRPMD simulations at 298.15 and 100 K. The high-frequency vibrational bands in the spectra are characterized using Fourier transform localized vibrational mode (FT-*ν_N_*AC) and interatomic distance histograms. At room temperature, the quantum effects are less significant, and the free energy profiles in the classical MD and the TRPMD simulations are very similar. However, the hydrogen bond between the hydroxyl–carboxyl bond is slightly stronger in TRPMD simulations. At 100 K, the quantum effects are more prominent, especially in the 2600–3600 cm^−1^, and the free energy profile slightly differs between the classical MD and TRPMD simulations. Using the FT-*ν_N_*AC and the interatomic distance histograms, the high-frequency vibrational bands are discussed in detail.

## I. INTRODUCTION

Vibrational spectroscopy has become an essential tool to understand the structure and dynamics of molecular systems. Vibrational spectra not only provide information about the presence and absence of chemical species but also their charge distributions and electrostatic interactions with their surrounding environment.^2^ There are several approaches to calculate the vibrational spectra. One of the simplest and computationally efficient approaches is normal mode analysis (NMA).^3^ NMA is based on the harmonic approximation, assuming the underlying potential energy surface to be locally quadratic about the equilibrium geometry. As a result, NMA suffers from several limitations and particularly it neglects the anharmonic effect, which limits applications to flexible systems with multiple conformers at finite temperatures or solute molecules interacting with diffusive solvents in the condensed phase systems. Several approaches based on post-harmonic approximations have been proposed, which are arguably most accurate in predicting the vibrational spectra.^4^ These approaches include vibrational second-order perturbation theory (VPT2),^5–7^ quasi-degenerate VPT2 theory,^8^ vibrational self-consistent field/virtual state configuration interaction (VSCF/VCI),^9–18^ VSCF + second-order perturbation theory (VSCF/PT2),^19,20^ VSCF + vibrational coupled-cluster theory (VSCF/VCC),^19,21^ and multi-configuration time-dependent Hartree (MCTDH) method.^22–24^ However, these post-harmonic methods are computationally expensive.

Alternatively, molecular dynamics (MD) simulation-based methods, such as Fourier transform of the dipole autocorrelation function (FT-DAC),^1,25–28^ is an efficient and general approach to calculate the vibrational spectra *on the fly*. Often in MD simulations, even though the whole system is treated quantum mechanically, while the Newtonian equation still guides the nuclear motion. The FT-DAC spectra obtained from such simulations do not include the nuclear quantum effects (NQEs) such as zero-point energy and quantum tunneling. In the literature, *ad hoc* harmonic correction terms have been proposed to correct the NQE artifacts.^1,25,29^ For instance, Ramirez *et al.* have shown the effectiveness of such a simple empirical correction.^29^ However, such empirical correction terms often only correct the post-simulation IR spectral intensities. At the same time, several studies showed the limitations of the classical MD simulations in simulating the nuclear quantum fluctuations, which are crucial in condense phase IR spectral calculations,^30–32^ hydrogen diffusion,^33,34^ and proton/hydride-transfer reactions.^31,35–39^

On the other hand, the more computationally expensive path-integral MD (PIMD)^40,41^ simulations have been developed to simulate the NQEs. The popular implementations are the centroid-based MD simulations (CMD)^42–44^ and the ring-polymer MD simulations (RPMD).^31,44–47^ The RPMD simulations are computationally efficient than the CMD simulations. The RPMD simulations originated from the imaginary time of Feynman's path-integral formalism of quantum statistical mechanics. They are a direct extension of the Newtonian mechanics in the imaginary timescale using classical MD in the extended phase space of the ring polymer.^41,48^ However, the RPMD simulations suffer from spurious peaks in the spectrum, when the physical peaks are in resonance with the internal modes of the ring polymer.^31^ This artifact can be corrected with an internal mode thermostat in MD simulations, so-called the thermostated RPMD method.^49,50^ Recently, Yu and Bowman^51^ calculated the vibrational spectra of ^+^H(H_2_O)_3_ and ^+^H(H_2_O)_4_ with thermostated ring-polymer molecular dynamics (TRPMD)^49,50^ simulation. In their work, they addressed the spurious peak problem by using an internal thermostat, i.e., path-integral Langevin equation (PILE) or generalized Langevin equation (GLE).^50^ Between these two thermostats, the GLE thermostat is more accurate for high-frequency vibrational bands but maybe less accurate for low-frequency ones than the PILE thermostat.

**FIG. 1.**
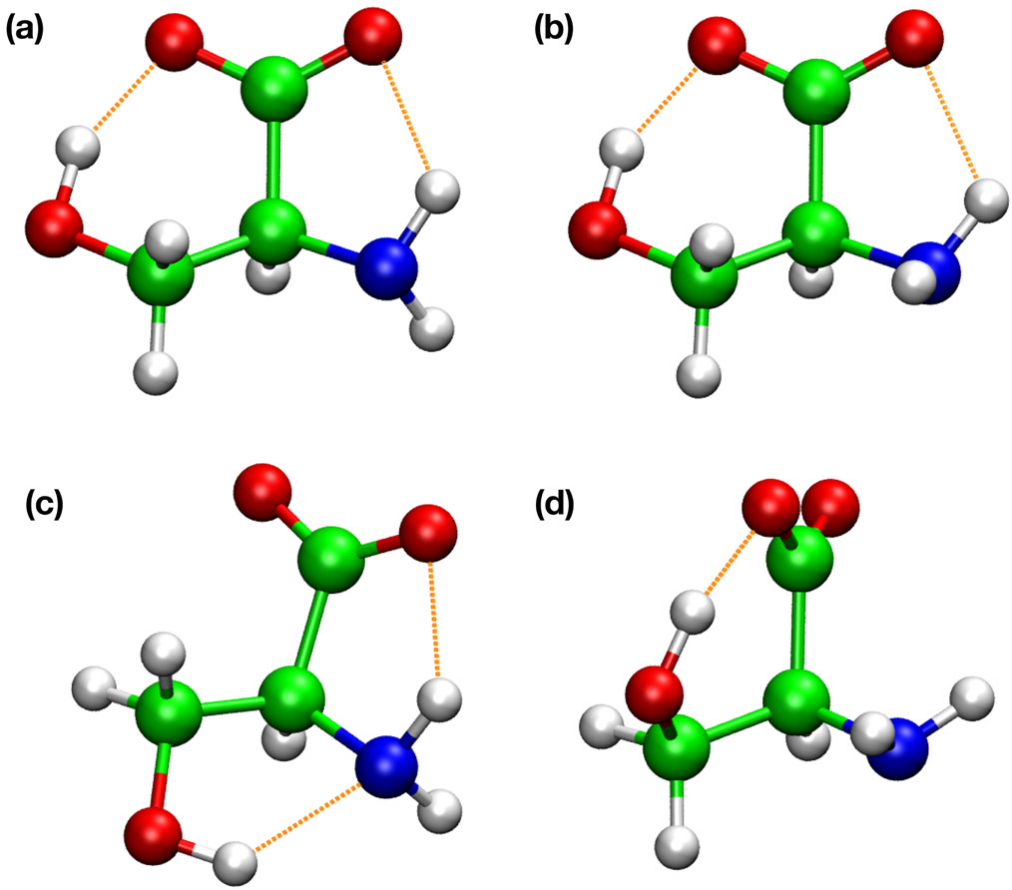
Four lowest energy conformations of deprotonated serine. (a) Conformer-I; (b) conformer-II; (c) conformer-III; and (d) conformer-IV. Conformations I, II, III have been identified in Ref. 59. Our recent work identified another minimum (conformer-IV). Their structural and energetic properties were summarized in Table I.

In our earlier work,^1^ we calculated the FT-DAC spectra of deprotonated serine from a classical MD simulation with the third-order density-functional tight-binding (DFTB3) method and corrected the IR intensities with an empirical *Q_HC_* term.^29^ At room temperature, the vibrational spectrum from the classical MD was able to reproduce the critical feature of the experimental infrared multiple photon dissociation (IRMPD) spectrum of serine (Fig. 1). We also noticed that the experimental spectra were more broadened than the FT-DAC spectra from classical MD simulations, and this has postulated that this is due to the neglected NQEs. In this study, we will address the effects of NQEs on the vibrational spectra of deprotonated serine with TRPMD simulations and explore potential limitations of the FT-DAC based on TRPMD simulations with DFTB3.

## II. COMPUTATIONAL DETAILS

### A. Classical molecular dynamics simulations

IR spectroscopy calculations based on the classical FT-DAC function have been described in detail in our previous work.^1,25^ Briefly, the MD simulation is equilibrated for 50 ps with a time step of 0.5 fs at a specific temperature (100.00 or 298.15 K) with Langevin dynamics. The following production run was carried out for ∼32 ps, sampling the microcanonical ensemble (NVE) with the velocity-Verlet integrator. The positions (${\vec{r}}_{i}$) and the Mulliken charges (*q_i_*) for atom *i* at each step were printed out and the dipole moment ($\vec{\mu }$) at each step was calculated using the following equation:

$$\vec{\mu }=\sum_{i=1}^{n}q_{i}{\vec{r}}_{i}.$$ (1)

Then, the classical FT-DAC function was calculated by the following equation:^52,53^

$$I_{cl}=\frac{1}{2\pi }\int_{-\infty }^{\infty }dt\;\text{exp}\;\left[-i\omega t\right]\langle \vec{\mu }(0)\cdot \vec{\mu }(t)\rangle .$$ (2)

The IR absorption coefficient *α_QC_* was calculated with

$$\alpha_{QC}(\omega )=\left[\frac{4\pi^{2}\omega }{3\text{Vℏcn}(\omega )}\right]\left(1-\text{exp}\;\left(-\beta \hbar \omega \right)\right)Q_{QC}(\omega )I_{cl}(\omega ),$$ (3)

where *V* is the sample volume, ℏ is the Plank's constant, *c* is the speed of light, and n(ω)≃1 is the refractive index of the medium. Here, a quantum correction term *Q_QC_* is included to approximately account for the quantum effects on nuclear motion. The simple harmonic correction term *Q_HC_* [Eq. (4)] proposed by Ramirez *et al.*^29^ was applied as previously described,^1,25^

$$Q_{HC}=\frac{\beta \hbar \omega }{1-\text{exp}\;\left[-\beta \hbar \omega \right]}.$$ (4)

The final IR absorption coefficient was given by

$$\alpha_{QC}(\omega )=\left[\frac{4\pi^{2}\omega }{3\text{Vℏcn}(\omega )}\right]\beta \hbar \omega I_{cl}(\omega ).$$ (5)

The IR spectra for deprotonated serine at 298.15 and 100 K have previously been reported and included here for comparison.^1^

### B. Thermostated ring-polymer molecular dynamics (TRPMD) simulations

TRPMD simulations were carried out with the *i-PI* software^54,55^ interfaced with DFTB+.^56^ First, the equilibrium simulations were to run for 40 ps. Then, the following 50 snapshots sampled every 1000 steps were used as the initial structures for the TRPMD simulations. The simulations were coupled to generalized Langevin equation (GLE) thermostats.^50^ For each temperature, 50 independent simulations were run for ∼32 ps with 32 beads at 100 K and 16 beads at 298.15 K. We followed the similar protocol by Yu and Bowman to calculate the FT-DAC spectra.^51^ The Mulliken charge was recorded at every time step and the value of for the dipole moment at each step was calculated according to the following equation:

$$\vec{\mu }=\frac{1}{n_{\mathrm{beads}}}\sum_{j=1}^{n_{\mathrm{beads}}}{\vec{\mu }}_{j},$$ (6)

where *j* indicates the *j*th replica (bead) of the ring polymer. Then, the IR spectra were calculated following Eq. (3). Note that no extra empirical quantum correction was included. The intensities in all spectra were normalized by setting the maximal intensity in the range from 600 to 3600 cm^−1^ to 1.0.

### C. 2D potential of mean force simulations of serine

Umbrella sampling simulations were performed to obtain the 2D potential of mean force (2D-PMF) with the biased potentials applied along with two reaction coordinates defined by the dihedral angles of C_*α*_–C_*β*_–O_*hydroxyl*_–H_*hydroxyl*_ and C_*carboxyl*_–C_*α*_–C_*β*_–O_*hydroxyl*_. The classical MD and TRPMD simulations were carried out with CHARMM and i-PI solver, respectively. Both the simulations (2D-PMF) were performed with the DFTB3 theory for 100 ps for each window, which were separated by 5° alongside the reaction coordinates. In both simulations, the temperature was maintained either at 100 or 298.15 K. For the classical MD simulations at 100 K, the biased potential [$E_{\mathrm{bias}}=k\times (1-\text{cos}(\theta -\theta_{o}))$—the dihedral in radian] of 75 and 65 kcal/mol, respectively, whereas at 298.15 K, 150 kcal/mol, and 125 kcal/mol potentials were used alongside the two reaction coordinates. In TRPMD simulations, a bias potential with a force constant 200 kJ/mol [$E_{\mathrm{bias}}=\frac{1}{2}\times K\times {\left(\theta -\theta_{o}\right)}^{2}$—the dihedral in degrees] was used alongside the reaction coordinates. The PMF was constructed with the weighted histogram analysis method.^57,58^ The convergence was monitored by examining the PMFs based on the first 50 ps and the last 50 ps trajectories, and the error was estimated to be less than 0.1 kcal/mol.

### D. Characterization of the spectral peaks

The peak characterization in the FT-DAC spectra is not as straightforward as the NMA method. Several methods have been proposed to characterize the FT-DAC spectra peaks (see detailed discussions in Ref. 26). Among them, Nishimura *et al.* implemented the Fourier transform of the localized vibrational mode autocorrelation (FT-*ν_N_*AC) function to characterize the vibrational bands in methanol dimer.^26^ This method is well suited for the characterization of the spectral peaks, especially for small molecules, and it was adopted in our previous work^1^ and the current work. Briefly, the Cartesian coordinates of the molecules are extracted at each time step from MD simulations. Then, the internal coordinates of each internal mode *ν_N_* are calculated. For instance, the internal coordinates of the O–H stretch peak are obtained as the distance between the oxygen and hydrogen atoms. Power spectra of these internal modes *ν_N_* are obtained by the Fourier transformation of the autocorrelation function $\left\langle {\vec{\nu }}_{N}(0)\cdot {\vec{\nu }}_{N}(t)\right\rangle$. The obtained power spectra might not provide the intensities of the individual vibrational modes but the location of the localized vibrational modes.

## III. RESULTS AND DISCUSSION

First, we present the results of conformational space analysis and FT-DAC vibrational spectra of deprotonated serine molecules with classical MD and TRPMD simulations at 298.15 and 100 K. This section also describes the influence of NQEs on the structural and energetic properties of serine molecules at two different temperatures. Finally, with the help of the Fourier transform of the localized vibrational mode (*ν_N_*) autocorrelation function (FT-*ν_N_*AC) and interatomic distance histograms, we tried to understand the reasons behind the difference in the FT-DAC spectra from classical MD and TRPMD simulations.

### A. The free energy landscape of serine

**FIG. 2.**
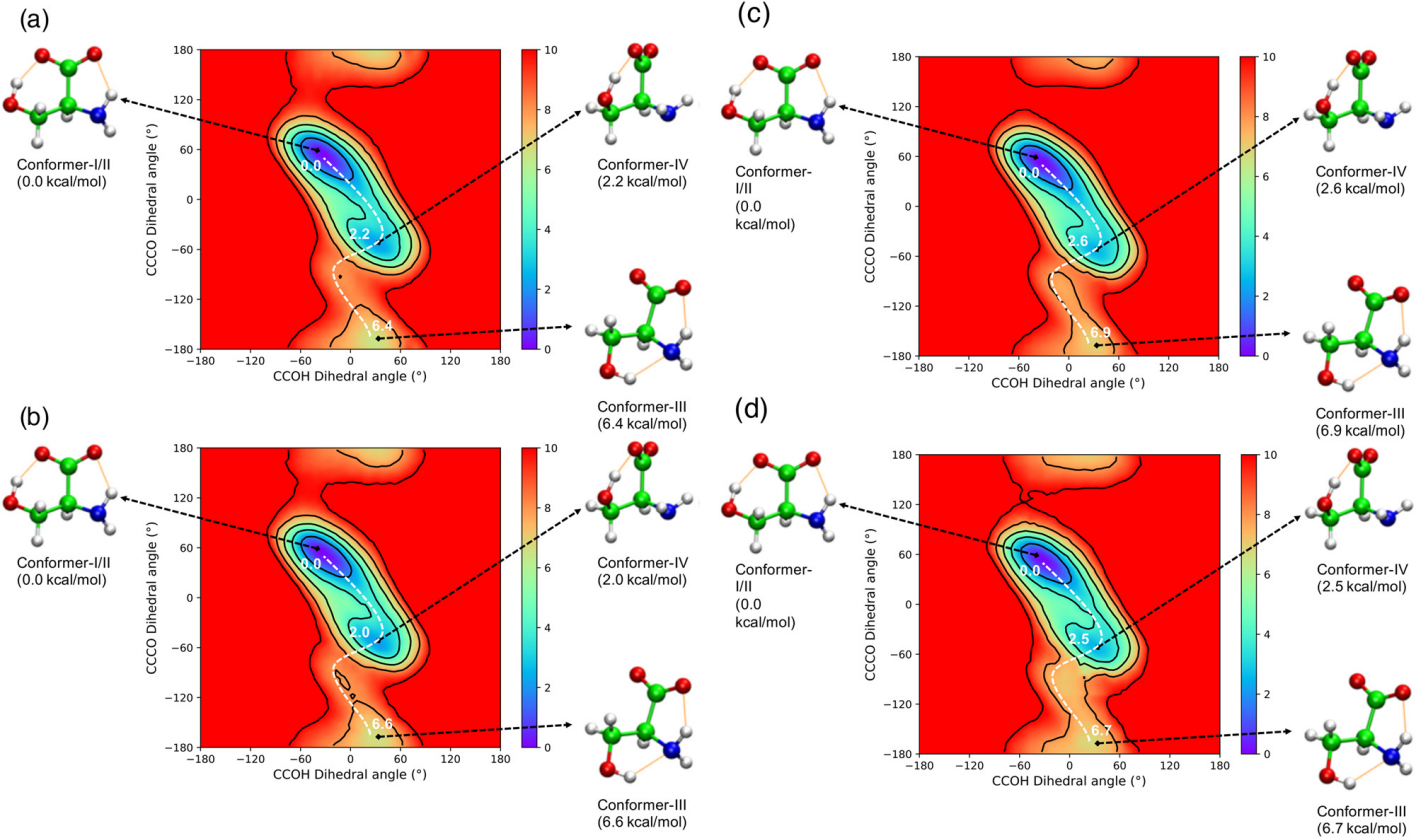
2D-PMF of serine conformations (a) classical MD simulations, (b) TRPMD simulations at 298.15 K and (c) classical MD simulations, and (d) TRPMD simulations at 100 K. The contour plot showing the zoomed free energy landscape in the range of 0–10 kcal/mol.

The four minimal energy structures of deprotonated serine optimized with DFTB3 presented in Fig. 1 and their energetic and structural properties were summarized in Table I. Conformers I–III have been reported by Oomens *et al.*^59^ while conformer-IV was recently identified (in Ref. 1) In our earlier study,^1^ we analyzed the conformational space of the deprotonated serine structure with classical MD simulations at 298.15 K. To study the influence of NQEs on the structural and energetic properties of serine, the 2D-PMF simulations were performed at 298.15 and 100 K with classical MD and TRPMD simulations. Table II presents the relative free energies of the deprotonated serine conformations and the transition states (TS-I & II) between each conformation. Figure 2 presents the contour plots of the deprotonated serine conformational space (with classical MD and TRPMD) at 298.15 and 100 K.

**TABLE I.** The structural and energetic properties of serine conformers I–IV at the DFTB3 level. The conformations are characterized by the two torsional angles. The energetic properties were in kcal/mol.^1^

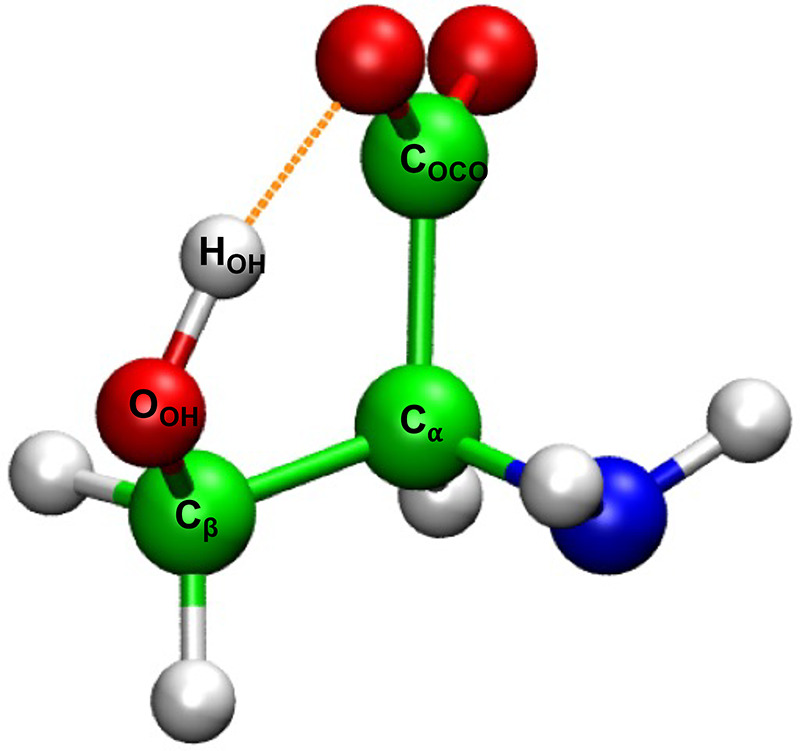			
	Torsional angles		
	C_*α*_–C_*β*_–O_OH_–H_OH_	C_OCO_–C_*α*_–C_*β*_–O_OH_	ΔE
Conformer-I	−35	+55	0.3
Conformer-II	−35	+55	0.0
Conformer-III	+35	−170	7.0
Conformer-IV	+35	−55	2.4

**TABLE II.** The classical and TRPMD relative free energy difference (ΔG) between the three conformations of deprotonated serine at 298.15 and 100 K in kcal/mol.

	Classical MD (kcal/mol)		TRPMD (kcal/mol)	
	298.15 K	100 K	298.15 K	100 K
Conformer-I/II	0.0	0.0	0.0	0.0
Conf-IV to I/II (TS-I)	1.4	0.9	1.5	1.3
Conformer-IV	2.2	2.5	2.2	2.5
Conf-III to IV (TS-II)	1.8	1.3	1.8	0.8
Conformer-III	6.4	6.9	6.6	6.7

At 298.15 K, there was no significant difference in the relative free energies of the conformations and transition states calculated with the classical MD and TRPMD simulations [Figs. 2(a) and 2(b)]. The TRPMD simulations predicted 0.1 and 0.2 kcal/mol (Table II) higher free energy difference for TS-I and Conformer III, respectively. At 100 K, the NQEs are expected to be more significant than at room temperature [Figs. 2(c) and 2(d)]. The relative energies of the conformations were very similar in both classical MD and the TRPMD simulations. However, the relative energies of the transition states were slightly different. At TS-I, the TRPMD simulations predicted 0.4 kcal/mol higher relative free energy than that of the classical MD simulations, whereas at TS-II, 0.5 kcal/mol lower (Table II). It was worth mentioning that the reaction coordinate chosen in this study is two torsional degrees of freedom. The NQEs on them are not clear, and we will provide more detailed analyses below.

### B. FT-DAC spectra of serine

#### 1. 600–1800 cm^−1^ region

**FIG. 3.**
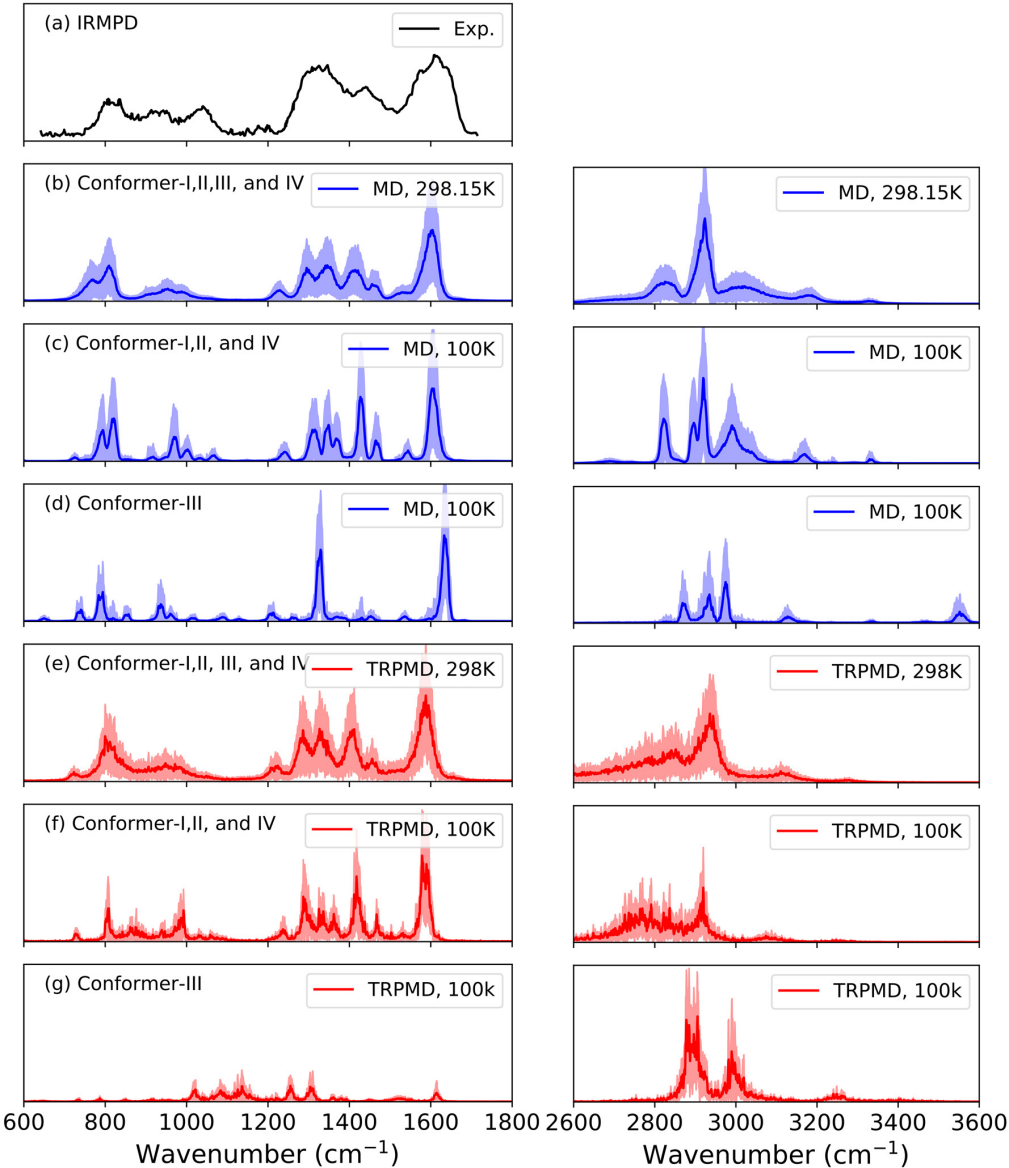
FT-DAC spectra of deprotonated serine with classical MD and TRPMD simulations at 298.15 and 100 K. FT-DAC spectra were normalized to the highest peak in their respective spectra. The experimental spectrum is shown in black (digitalized from the data in Ref. 59), DFTB3/FT-DAC spectra from classical MD simulation are shown in blue (standard deviation is presented as a blue shaded region), and DFTB3/FT-DAC spectra from TRPMD simulation are shown in red (standard deviation is presented as a red shaded region). DFTB3/FT-DAC spectra from classical MD simulation are taken from Ref. 1. Reproduced with permission from Inakollu and Yu, J. Comput. Chem. **39**, 2067 (2018). Copyright 2018 Wiley Periodicals, Inc.

Figure 3 presents the FT-DAC spectra of deprotonated serine from the experimental,^59^ classical MD,^1^ and TRPMD simulations at 298.15 and 100 K in 600–1800 cm^−1^ region.

In our earlier work,^1^ we found that at 298.15 K, conformers-III and IV can cross the barriers (TS-I and II) and converge to conformer-I/II. Occasionally, conformer-I/II also moved to conformer-IV. Overall at room temperature, conformer-I/II were the major ensemble structures in all the simulations. In this region, the FT-DAC spectra from both the classical MD and TRPMD simulations were very similar to each other, including both the position and shape of the peaks [Figs. 3(b) and 3(e)]. Whereas at 100 K, conformation III of serine did not cross the energy barrier (TS-II in Table II). The FT-DAC spectra obtained from the conformer-I/II and IV were presented in Figs. 3(c) and 3(f), and the FT-DAC spectra from conformer-III were showed in Figs. 3(d) and 3(g). Both the classical MD and TRPMD simulations predicted similar FT-DAC spectral peak positions for conformation I/II and IV. However, the shape of the spectral peaks slightly differed from each other. Notably, in the 700–1100 cm^−1^, the TRPMD/FT-DAC spectra were broadened than those by classical MD/FT-DAC.

The FT-DAC spectra of conformer-III at 100 K were significantly different. The spectra obtained from the conformation III with the classical MD and TRPMD simulations were very different from each other [Figs. 3(d) and 3(g)]. In the FT-DAC spectra from the classical MD simulations, most of the spectral peaks were at low intensity, except the peaks near 1610 and 1300 cm^−1^. The 1610 and 1300 cm^−1^ peaks are the attributes of C=O stretch and OCO symmetric stretch peak, respectively.^1^ Whereas in TRPMD, all the peaks in 600–1800 cm^−1^ spectral region were low intensity, including the peaks near 1610 and 1300 cm^−1^.

#### 2. 3000–3600 cm^−1^ region

Since the quantum effects are expected to be more prominent in the higher frequency regions at lower temperatures, we compared the FT-DAC spectra from the classical MD and TRPMD simulations in 3000–3600 cm^−1^ region. At 100 K, the spectra calculated with the classical MD and TRPMD simulations were significantly different from each other [Figs. 3(e) and 3(g)]. In the spectra of conformers I, II, and IV at 100 K, the peak near 2800 cm^−1^ in the classical MD simulations was broadened to the lower-frequency region in the TRPMD simulations, and the 3000 cm^−1^ disappeared as well. Both the simulations predicted the peaks near 2900 cm^−1^ and 3250 cm^−1^ in a similar location, but the shape and intensities of these peaks were dissimilar. The spectra obtained from the conformation III at 100 K from both simulations also differ. Classical MD simulations predicted a peak near 3550 cm^−1^, which was missing in the TRPMD simulations. The peak near 3150 cm^−1^ in the classical MD simulation was shifted to 3250 cm^−1^. Finally, the two peaks near 2900 cm^−1^ were merged in the TRPMD simulations.

Even at 298.15 K, the spectra from the classical MD and TRPMD simulations were different from each other. The broad peak near 3000 cm^−1^ in the classical MD simulation was disappeared in the TRPMD simulations, and the peak near 2800 cm^−1^ was significantly broadened to lower frequencies. However, the peaks near 2900 and 3250 cm^−1^ were similarly predicted by both the simulations. To understand the observed differences, spectra characterization and geometric analysis were carried out.

### C. Spectral characterization in 2600–3600 cm^−1^ region

**FIG. 4.**
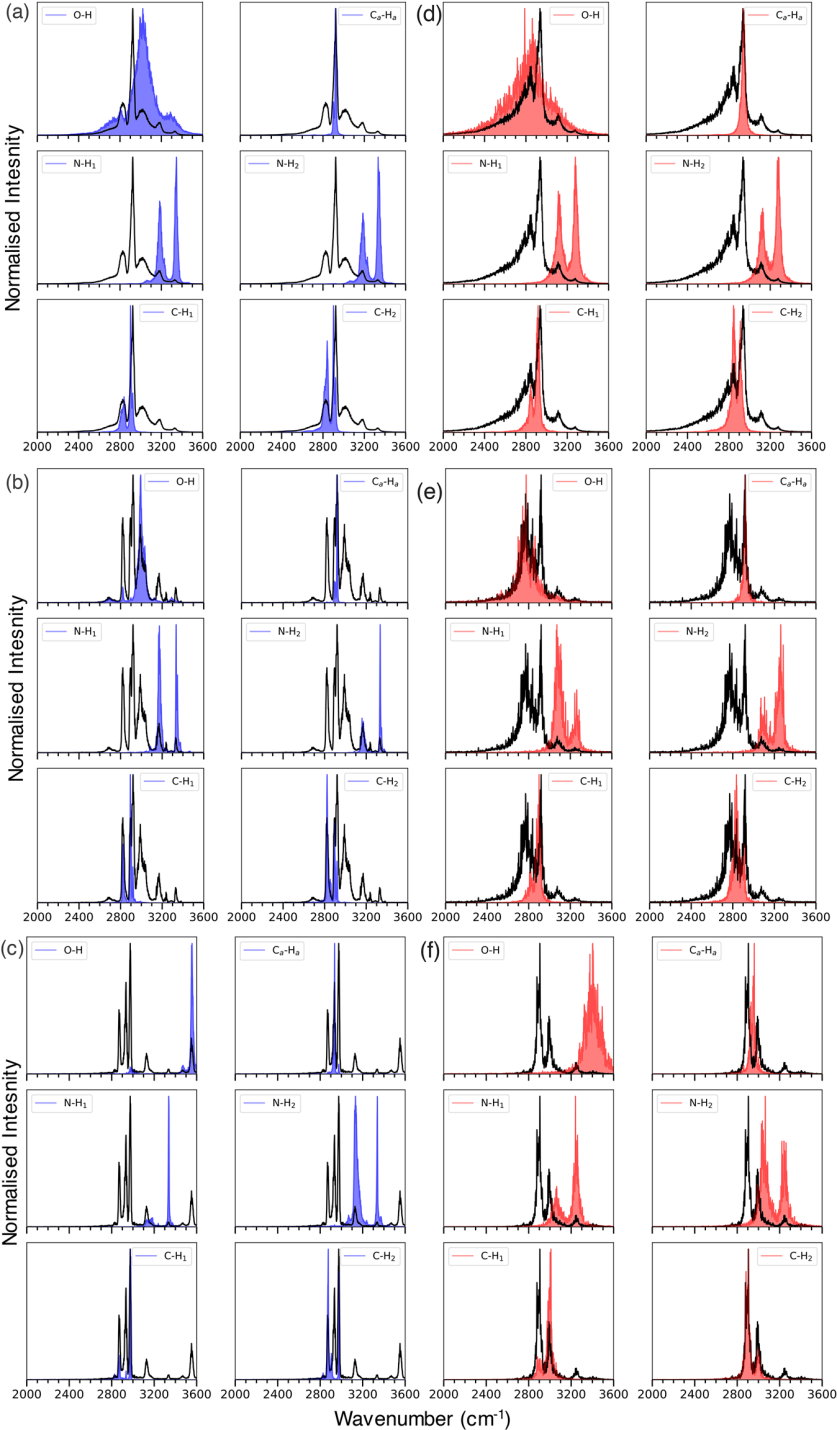
Characterization of FT-DAC spectra of deprotonated serine. FT-*ν_N_*AC method was used to interpret the FT-DAC spectral features obtained from classical MD and TRPMD simulations at 298.15 and 100 K in 2600–3600 cm^−1^ region. a, b, and c present the FT-DAC spectra and FT-*ν_N_*AC (blue shade) of conformer-I–IV (298.15 K), conformer-I/II/IV (100 K), and conformer-III (100 K), respectively, obtained from the classical MD simulations; d, e, and f present the FT-DAC spectra and FT-*ν_N_*AC (red shade) of conformers I–IV (298.15 K), conformer-I/II/IV (100 K), and conformer-III (100 K), respectively, obtained from the TRPMD simulations.

**FIG. 5.**
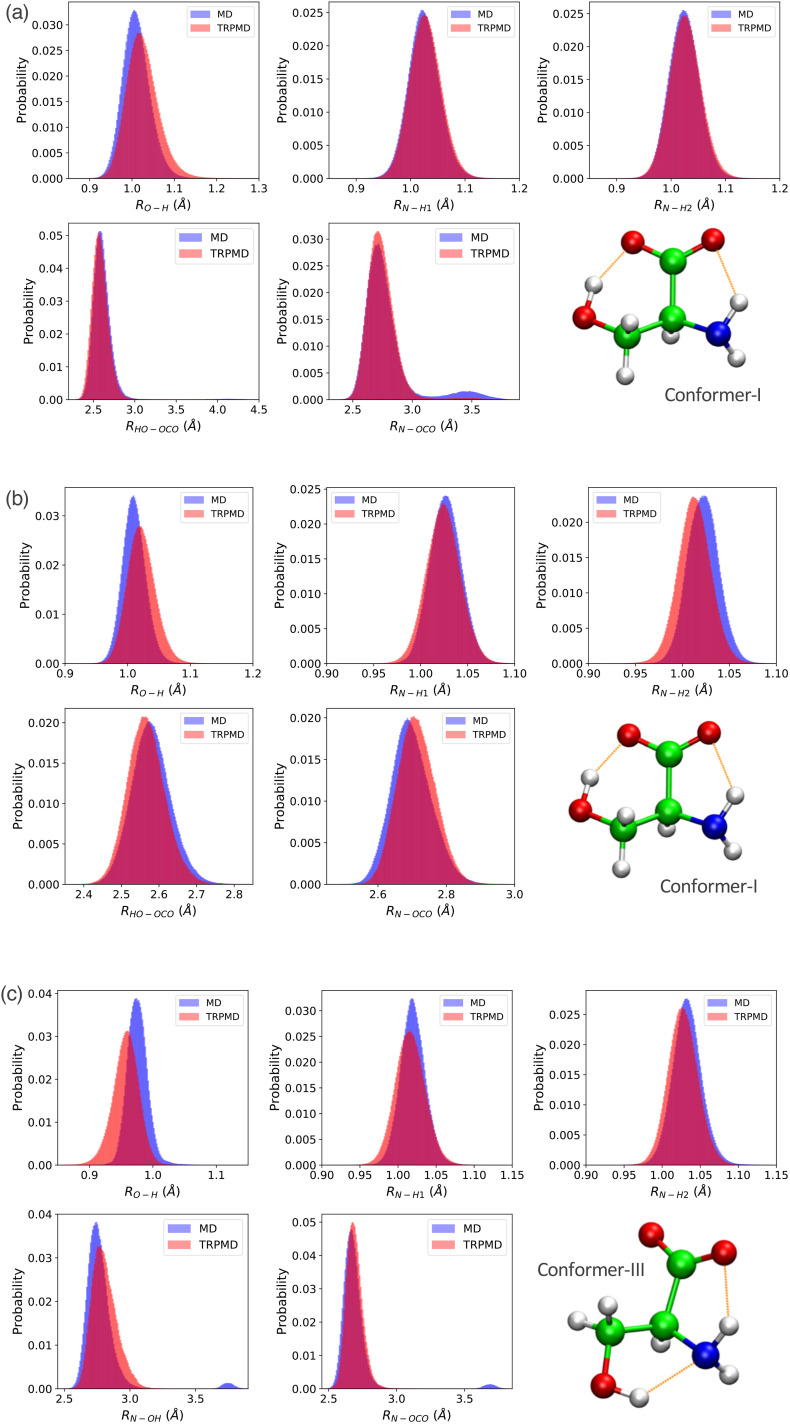
Histograms of interatomic distance in the classical MD and TRPMD simulations at 298.15 and 100 K of deprotonated serine. (a) Conformer-I–IV (298.15 K), (b) conformer-I/II/IV (100 K), and (c) conformer-III (100 K); interatomic distance distribution in the classical MD simulations is presented in blue and TRPMD simulations are in red. The interatomic distance between the groups is presented in the x-axis.

In this section, we used the Fourier transform of the localized vibrational mode autocorrelation (FT-*ν_N_*AC) function for the spectral characterization.^1,26^ Figure 4 presents the FT-DAC spectral peak characterization of deprotonated serine in 2600–3600 cm^−1^ region with the classical MD and TRPMD simulations, respectively. To support the FT-*ν_N_*AC method, we have also analyzed the bond lengths and interatomic distance distributions and presented in Fig. 5 in the Appendix. The FT-*ν_N_*AC method was able to characterize all the peaks, i.e., stretch peaks of C–H, N–H, and O–H, in this region, and able to predict the spectral similarities and differences accurately.

#### 1. At 298.15 K

As mentioned earlier, the most noticeable difference between the spectra of deprotonated serine at 298.15 K was the peaks near 3000 and 2800 cm^−1^ [Figs. 4(a) and 4(d)]. The difference in the spectral features was mostly due to the shift in the hydroxyl stretch peak position. In the classical MD simulations, the hydroxyl stretch peak was predicted near 3000 cm^−1^, whereas, in the TRPMD simulations, it was near 2800 cm^−1^ and fused with the C–H stretch peak. Interestingly, due to the stronger internal hydrogen bond network, the hydroxyl stretch peak was red-shifted approximately by >600 cm^−1^ from its usual frequency location (e.g., ∼3600 cm^−1^) in both the classical MD and TRPMD simulations.

In support of the FT-*ν_N_*AC, the interatomic distance distributions (Fig. 5 a in the Appendix) of the oxygen and hydrogen of the hydroxyl group in the TRPMD simulations were slightly broadened than that of the classical MD simulations. Furthermore, the histograms of the hydroxyl–carboxyl (HO–OCO) and carboxyl–amine (OCO–NH_2_) groups show that the distance between these groups was distributed in two different populations, especially the OCO–NH_2_ groups. This heterogeneous distribution indicates that at 298.15 K, the ensemble structure of serine includes both conformer-I/II and the conformer-IV. Apart from the dissimilarities in the spectra of the classical MD and TRPMD simulations, FT-*ν_N_*AC also predicted the similarities, such as the N–H and C–H stretch peaks in their respective spectra.

#### 2. At 100 K

At 100 K, the FT-DAC spectra calculated from the conformations I/II/IV predicted the hydroxyl stretch peak at 3000 to 2800 cm^−1^ in classical MD and TRPMD simulations, respectively [Figs. 4(b) and 4(e)]. This observation was supported by the bond-length distribution [Fig. 5(b) in the Appendix]. Unlike the spectra at 298.15 K, the heterogeneous population of the OCO–NH_2_ groups was not observed at 100 K in both the simulations. In the TRPMD simulations, the interatomic distance of HO–OCO was slightly shorter, and OCO–NH_2_ was slightly longer than that of the classical MD simulations. The increased interatomic distance in the hydroxyl bond and decreased interatomic distance between the HO–OCO groups indicate that there was a stronger hydrogen bond interaction between the HO–OCO in the TRPMD simulations.

Interestingly, both the N–H stretch peaks in the TRPMD simulations were red-shifted by ∼70 and ∼100 cm^−1^, in comparison with classical MD simulations [Figs. 4(b) and 4(e)]. This red shift indicates that there was a stronger hydrogen bond interaction between the carboxyl and amine groups. As mentioned earlier, a slightly increased TS-I energy barrier between the conformer-I/II and IV was observed in the TRPMD simulations. To move from conformer-I/II to IV, the serine molecule has to break the intramolecular hydrogen bond between the carboxyl and amine groups. Therefore, the increased TS-I energy barrier in TRPMD simulations might arise from the stronger hydrogen bond interaction between the carbonyl and the amine group.

As mentioned earlier, at 100 K the simulations starting from conformer-III retained their initial conformation in both the classical MD and TRPMD simulations. Compared to the simulations from conformer-I/II/IV, the hydroxyl bond distance was shorter in the conformer-III simulations. The hydroxyl bond average distance in conformer-III was shifted by 0.25 and 0.35 Å from conformer-I/II/III in classical MD and TRPMD simulations, respectively [Figs. 5(b) and 5(c) in the Appendix]. The decreased hydroxyl bond length resulted in the higher frequency of the hydroxyl stretch peak in the FT-DAC spectra. The hydroxyl peak in the FT-DAC spectra of conformer-III was around 3550 cm^−1^ region, whereas it was ∼3000 cm^−1^ in the conformer-I/II/IV spectra at both temperatures. In the case of the N–H stretch peak, the conformer-III showed a similar trend as the conformer-I/II/IV at 100 K, i.e., red shift of 25 and 100 cm^−1^ in the amine peaks of the TRPMD FT-DAC spectra. Another interesting observation from the FT-DAC spectra of conformer-III at 100 K is that in the classical MD simulations, the amine–hydroxyl and amine–carboxyl interatomic distance were distributed in two different populations. In contrast, in TRPMD simulations, this feature was not observed. However, the histograms of TRPMD simulations of these interatomic distances were slightly broadened and screwed to higher distances. The heterogeneous population in the histogram of conformation III represents the trail it made to reach conformation IV.

## IV. CONCLUSIONS

In this study, we have investigated the influence of the NQEs on the vibrational spectra of the deprotonated serine molecule. We have compared the similarities and differences between the FT-DAC spectra calculated from the classical MD and the TRPMD simulations at 298.15 and 100 K. In addition to the FT-DAC spectra, we have analyzed the conformational space of serine with classical MD and TRPMD simulations at two temperatures. Both the spectra from classical MD and TRPMD simulations were able to capture the key feature of its experimental spectra in 600–1800 cm^−1^ region. The FT-*ν_N_*AC method and the interatomic distance histograms were used to interpret the spectroscopic features in 2600–3600 cm^−1^ region.

At 298.15 K, despite the difference in the hydroxyl stretch peak location and distribution, we did not find a significant difference in the FT-DAC spectra and the free energy profile between the classical MD and TRPMD simulations. At 100 K, minor NQEs were observed. First, a minor effect was observed on the free energy barriers between different conformers in the PMF simulations. Apart from the hydroxyl stretch peak shift, the N–H stretch peaks were red-shifted in the TRPMD simulations, indicating a stronger hydrogen bond interaction between the carboxyl bond and amine groups. This stronger hydrogen bond interaction explains the reason behind the slightly higher energy barrier of TS-I.

In summary, current vibrational spectra calculations with FT-DAC based on the TRPMD simulations with DFTB3 have shown to be able to capture NQEs in the deprotonated serine, and such effects can be correlated with observed structural properties. The remaining difference observed between the calculated spectra and the experimental spectra are partly due to the potential limitations of the quantum mechanical (QM) method, as noted in our previous study.^1^ This can be further improved by applying a post-simulation correction of the FT-DAC spectra with single point vibrational frequency calculations with a high-level method.^1,60^ Also, the dipole moments are derived based on Mulliken charges, routinely used in DFTB3 based simulations.^1,25,61,62^ However, it is known to have limitations in reproducing the dipole moments. Refinement of the charge calculation can further improve the DFTB3 based FT-DAC calculations and will be explored in our future work.^62–64^

## Data Availability

The data that support the findings of this study are available from the corresponding author upon reasonable request.

## APPENDIX: INTERATOMIC DISTANCE HISTOGRAMS

To understand the FT-DAC spectra features in 2600–3600 cm^−1^ in the classical MD and TRPMD simulation, we have also compared the interatomic bond histograms (Fig. 5). The peaks in the higher frequency region are C–H, N–H, and O–H stretch peaks. From the spectral peak characterization with the FT-*ν_N_*AC method, the major deviations between the classical MD and TRPMD simulation spectral features are majorly from the N–H and O–H stretch modes. Figure 5 presents the interatomic distance of the atoms, which are involved in the internal hydrogen-bonding network, e.g., O–H, N–H_1_, N–H_2_, OCO–OH, OCO–NH, and HN–OH.
